# Fructose as a novel nutraceutical for acetaminophen (APAP)-induced hepatotoxicity

**DOI:** 10.20517/mtod.2023.28

**Published:** 2023-10-29

**Authors:** Kyle Rodrigues, Rawdat Hussain, Sarah Cooke, Gary Zhang, Deqiang Zhang, Lei Yin, Xin Tong

**Affiliations:** 1 Department of Molecular & Integrative Physiology, University of Michigan Medical School, Ann Arbor, MI 48105, USA.; 2 Caswell Diabetes Institute, University of Michigan Medical School, Ann Arbor, MI 48105, USA.

**Keywords:** Acetaminophen toxicity, fructose, ChREBPα, FGF21

## Abstract

Acetaminophen (APAP) is the most widely used analgesic in the world. APAP overdose can cause severe hepatotoxicity and therefore is the most common cause of drug-induced liver injury. The only approved treatment for APAP overdose is N-acetyl-cysteine (NAC) supplementation. However, the narrow efficacy window of the drug severely limits its clinical use, prompting the search for other therapeutic options to counteract APAP toxicity. Recent research has pointed to fructose as a novel nutraceutical for APAP-induced liver injury. This review summarizes the current understanding of the molecular mechanisms underlying APAP-induced liver injury, introduces how fructose supplementation could prevent and treat APAP liver toxicity with a focus on the ChREBPα-FGF21 pathway, and proposes possible future directions of study.

## INTRODUCTION

APAP (N-acetyl-para-aminophenol, paracetamol) is one of the most commonly used pain killers as a widely available over-the-counter medication^[1]^. APAP is a nonsteroidal anti-inflammatory drug (NSAID) that works by inhibiting cyclooxygenase (COX) in the brain, thereby enabling it to manage fever and alleviate pain^[2,3]^. It inhibits prostaglandin synthesis in the central nervous system^[4,5]^. APAP produces an antipyretic effect by acting directly on the hypothalamus^[6]^. Generally perceived as a very safe medication, APAP is consumed as acetaminophen by more than 60 million Americans on a weekly basis^[7]^.

APAP overdose is the most common cause of acute liver failure in the US, with around 30,000 patients admitted to hospitals every year, accounting for about 48% of acute liver failure diagnoses^[7,8]^. 29% of those patients undergo liver transplants, with a mortality rate of 28%^[7]^. Acetaminophen has become even more widespread since many products containing APAP have been sold and consumed in tandem with other medications, especially opioids and diphenhydramine^[9]^. 63% of unintentional overdoses of APAP occur in patients consuming the opioid/APAP combination, highlighting the ever-increasing risk of APAP liver injury as a silent killer^[7]^.

The amount of acetaminophen ingested determines the symptoms of APAP toxicity^[10,11]^. Mild poisoning may not cause any manifestations, while severe overdose could be life-threatening. Acetaminophen toxicity is typically divided into four stages. Stage one (the incubation period) encompasses the initial 24 h. In this stage, patients may be asymptomatic or have non-specific symptoms such as vomiting, abdominal pain, nausea, and loss of appetite^[12]^. These symptoms are reminiscent of the flu or a regular cold. Thus, the possibility of APAP toxicity being the root cause may be overlooked. Unfortunately, the treatment for APAP toxicity tends to be most efficient if the diagnosis is confirmed in the first stage^[7]^. During stage two, which is 24–48 h after ingestion, patients may develop hepatic toxicity or exhibit right-upper quadrant pain, and the patients’ renal function may also deteriorate^[13]^. It is possible that some of the non-specific symptoms from the first stage may disappear and the patient’s condition may improve^[7]^. About 72–96 h after ingestion, stage three starts with stage one symptoms returning along with elevated serum aspartate transaminase (ALT) and aspartate transferase (AST) levels (which could also be present in stage 2), and other comorbidities such as hypoglycemia, encephalopathy, lactic acidosis, and jaundice^[14]^. APAP is metabolized to a toxic metabolite called N-acetyl-p-benzoquinone imine (NAPQI) that begins to accumulate in the cell, leading to mitochondrial dysregulation^[15]^. In critical conditions, this stage poses the highest mortality rate due to multi-organ failure^[14]^. The final stage (stage four) transpires approximately 96 h after the third stage, and it usually lasts around 1–2 weeks, but it may also be longer depending on the severity of the overdose^[7]^. 70% of people recover completely within 3 months given proper treatment, while 1% to 2% of untreated patients develop critical hepatic failure, resulting in death 4 to 18 days after ingestion^[13]^.

## APAP ABSORPTION IN THE DUODENUM

When APAP is consumed orally, it is absorbed into the duodenum via passive diffusion^[16,17]^. APAP will move from the intestinal tract lumen across a mucosal membrane into the bloodstream by either direct diffusion through epithelial and endothelial cells or paracellular transport^[18,19]^. The primary location of absorption is the small intestine through the so-called “absorption window” of the duodenum and jejunum, although small (almost negligible) amounts of APAP are absorbed by the stomach^[16]^. The rate-determining step of the absorption of acetaminophen is gastric emptying (the process of the contents of the stomach moving to the duodenum)^[16]^. Once absorbed, APAP is metabolized in the liver. Large doses of the drug, however, can cause destruction of cellular microvilli, which decreases the surface area where absorption of extracellular compounds may occur^[17]^. Furthermore, large doses of APAP cause an oversaturation of efflux transporters in the body, also limiting APAP elimination in the gut^[17]^. Thus, when hepatotoxic doses of acetaminophen are ingested, its absorption period is prolonged.

## CURRENT MOLECULAR MODELS OF APAP-INDUCED LIVER TOXICITY

APAP metabolism primarily occurs in the liver. However, the majority (~ 90%) of APAP undergoes the phase II pathway in the liver, in which it goes through glucuronidation and sulfation by UDP-glucuronosyl transferases (UGT) and sulfotransferase (SULT), eventually being eliminated through urine^[20,21]^. A small portion of APAP is directly secreted through urine without being metabolized^[22]^. About 5%−10% of un-glucuronidated APAP is metabolized by CYP2E1 and CYP1A2 to generate the electrophilic reactive metabolite N-acetyl-p-benzoquinone imine (NAPQI)^[23]^, which is the main culprit of APAP toxicity. Previous studies have shown that *Cyp2e1* and *Cyp1a2* double-knockout mice displayed survival rates above 90% after being injected with APAP at doses up to 1200 mg/kg, highlighting the importance of CYP2E1 and CYP1A2 in the formation of NAPQI^[24]^. Under normal circumstances, NAPQI can be reduced by glutathione (GSH) to non-toxic mercapturate and cysteine compounds before excretion in urine^[20,25]^. Fasted mice or mice with low levels of endogenous GSH demonstrated increased severity of APAP-induced hepatic necrosis, whereas pretreatment of mice with precursors of GSH to elevate the endogenous GSH helped reduce liver damage^[26]^. However, if APAP overdose overwhelms the aforementioned detoxification pathways and depletes GSH to less than 30% of its normal level in the liver, NAPQI accumulates and forms covalent bonds with sulfhydryl groups on cysteine and lysine residues in the mitochondria of the hepatocytes^[23,27]^. The formation of those protein adducts in the mitochondria leads to oxidative damage and necrosis in hepatocytes^[28]^.

## CURRENT TREATMENT OPTIONS FOR APAP OVERDOSE

N-acetyl cysteine (NAC) is used to treat patients with high acetaminophen levels. If administered within 8 h of ingestion, NAC can be fully protective against hepatotoxicity^[29]^. NAC works through multiple routes: (1) promoting hepatic GSH synthesis^[30]^; (2) preventing covalent modifications of cellular proteins by NAPQI^[31]^; (3) scavenging reactive oxygen species (ROS) such as peroxynitrite^[32,33]^.

Although NAC can be beneficial for up to 24 h post overdose, multiple studies have shown that administration of NAC in the first 8–10 h is significantly more effective than late administration (16–24 h)^[34–36]^. Additionally, NAC administration can possibly lead to increased serum transaminases (ALT and AST) and also cause some adverse effects such as skin rash, allergic reaction, bronchospasm, hypotension, and even death^[37,38]^. Since the majority of APAP overdose cases are not diagnosed until much later than the ideal efficacy window of NAC, combined with the aforementioned side effects, efforts have been initiated to identify new pharmaceuticals for APAP overdose.

Fomepizole is also used to treat high acetaminophen levels. It works by inhibiting CYP2E1 and preventing the activation of JNK to help protect against mitochondrial dysfunction. Some, however, have raised suspicion regarding its success in clinical trials. In one study, fomepizole has been shown to increase serum ALT in healthy subjects, which could be deadly when combined with APAP overdose^[39]^. Others have suggested that fomepizole can be used in tandem with other treatments like NAC^[40]^. Calmangafodipir has also been cited to reduce the effects of APAP toxicity in combination with NAC^[41]^. Calmangafodipir mimics manganese superoxide dismutase, a protein that helps prevent mitochondrial injury. It was successfully used in Phase 2 trials for chemo-induced peripheral neuropathy and the trial also found reduced liver injury biomarkers such as ALT and INR. However, the sample size of patients was low and none of the patients from the study encountered any significant hepatotoxicity, so more research needs to be done to assess its efficacy^[41]^. Artificial intelligence methods are also being developed to study and predict drug-induced liver injury^[42]^.

## IMPACT OF NUTRITIONAL FACTORS ON APAP OVERDOSE

The severity of APAP hepatotoxicity has been shown to be affected by nutritional status, pre-existing liver disease, usage of alcohol, and consumption of other liver-metabolized drugs^[43,44]^. Among these factors, nutritional status can be targeted and manipulated to provide relief of APAP hepatotoxicity.

An individual’s diet and alcohol consumption play a major role in the extent of hepatotoxicity caused by APAP overdose. Another key factor in APAP-induced hepatotoxicity is narcotic use. Similar to alcohol, narcotics (or opioids) damage the liver and lead to a greater susceptibility to APAP overdose^[45]^. In addition, people with chronic pain who take several medications at the same time are more vulnerable to further liver damage. Studies conducted on rodents have shown that fasting and food restriction (such as a calorie-reduced diet) may exacerbate the extent of damage from APAP overdose^[45]^. Tsuchiya *et al*. showed that fasting and food restriction greatly increased the expression of CYP2E1 (cytochrome P4502E1) in mice and reduced liver glutathione content, thereby worsening APAP liver damage^[46,47]^. Additionally, a human study showed that healthy men who had been fasting for 38 h exhibited reduced clearance of therapeutically dosed chlorzoxazone (a compound metabolized by CYP2E1), indicative of the decreased activity of CYP2E1^[48]^. Furthermore, compared to normal controls, individuals with eating disorders have also shown decreased GSH synthesis, and a therapeutic dose of APAP could potentially be an overdose, complicating the liver’s ability to reduce the toxic metabolite NAPQI in the case of APAP-induced hepatotoxicity^[20]^.

**Figure 1.**
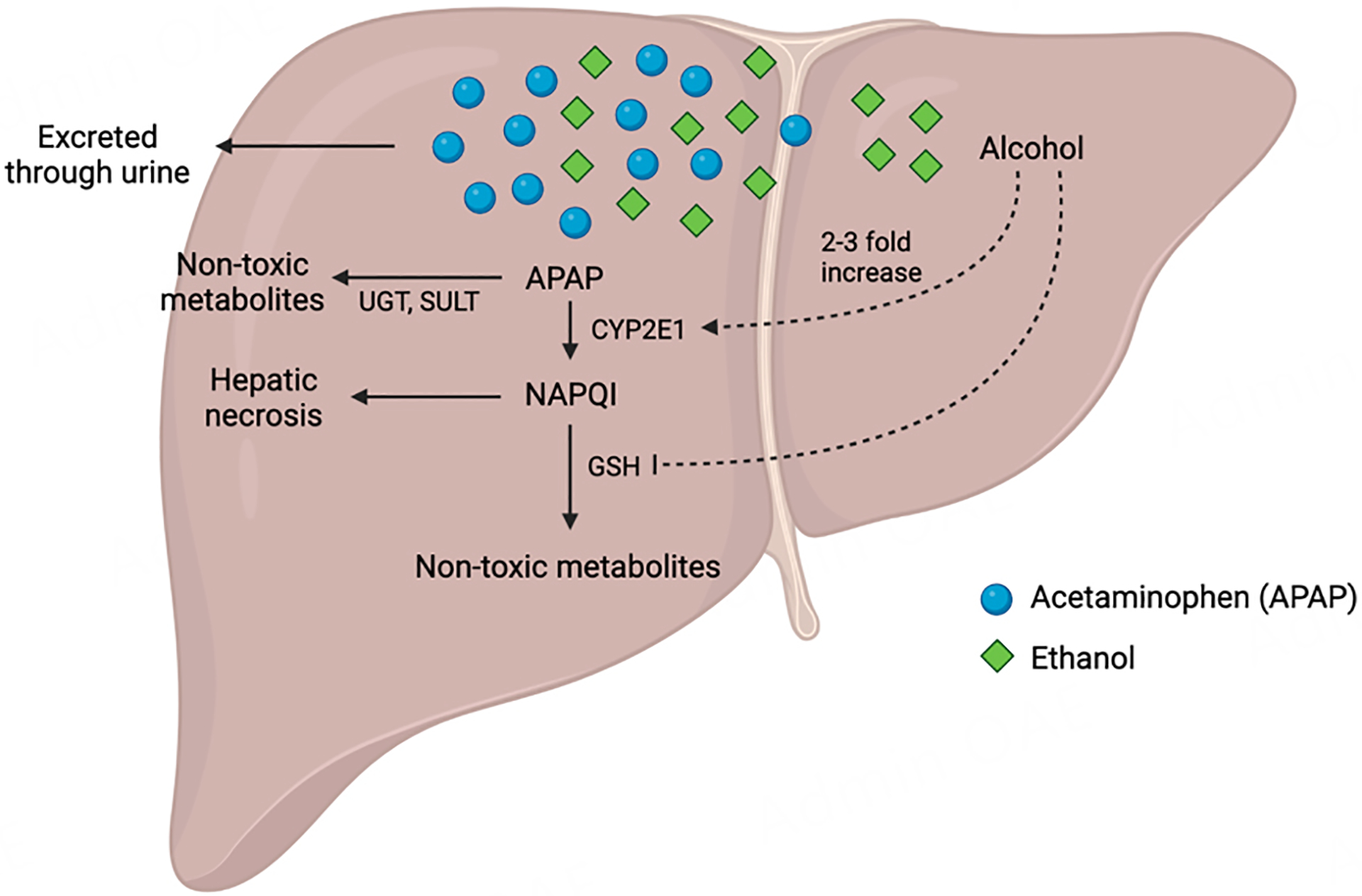
APAP metabolism in the liver and liver injury. APAP: Acetaminophen.

Alcohol also plays a major role in making patients more vulnerable to the hepatotoxic effects of acetaminophen by reducing the oxidative metabolism of the drug. Chronic alcoholics are prone to APAP hepatotoxicity not only following overdose, but also with its therapeutic use^[44]^. However, the influence of alcohol varies between chronic and acute consumption. Chronic use of alcohol leads to a short-term two- to threefold increase in hepatic cytochrome P4502E1 (CYP2E1)^[47]^ [Figure 1]. Chronic alcoholics have also been reported to express lower than normal plasma concentrations of the antioxidant GSH, which is able to detoxify the reactive metabolites. Experiments showed that hepatic levels of GSH quickly increased again when alcohol was cleared from test subjects^[49]^. Chronic alcoholics are at the highest risk during withdrawals when alcohol fails to counter APAP activation^[44]^. In contrast, acute consumption of ethanol protects animals against hepatotoxicity at doses as low as 2 mmol/L. This is due to the inhibition of the toxic metabolic activation of APAP, which alleviates liver damage^[44]^.

Most APAP goes through glucuronidation and sulfation by UGT and SULT to be eliminated as non-toxic metabolites. APAP is also metabolized to NAPQI, which can lead to liver necrosis and injury if unchecked or detoxified via GSH. Chronic alcohol use can significantly impact APAP-induced liver injury by increasing CYP2E1, which is critical for the metabolism of APAP, while depleting glutathione levels to reduce the liver’s ability to metabolize APAP into non-toxic compounds.

Together, nutritional status along with alcohol consumption can significantly affect an individual’s vulnerability to APAP hepatotoxicity.

## FRUCTOSE PROTECTION AGAINST APAP TOXICITY VIA THE CHREBP-FGF21 PATHWAY

Fructose is found naturally in fruits and vegetables, as well as in processed foods as additives such as table sugar and high-fructose corn syrup. The mean fructose consumption in the United States is 54.7g/day, with the primary intake being from sugar-sweetened beverages^[50]^. Although fructose intake can promote de novo lipogenesis and cause insulin resistance, a prelude to diabetes, non-alcoholic fatty liver disease (NAFLD), and obesity, recent studies have highlighted fructose as a potential antidote against APAP-induced hepatotoxicity^[51,52]^. Our own study also found that fructose ingestion could ameliorate APAP-induced hepatotoxicity. We further revealed that prompt fructose intake after APAP overdose could significantly mitigate liver damage through the activation of the ChREBP-FGF21 axis^[53]^. Based on literature and our report^[52,53]^, we propose that fructose could be utilized as a novel detoxification agent for drug-induced liver damage.

### Fructose intake on the APAP-metabolizing enzymes and its detoxification

The expression levels of CYP2E1 and CYP1A2- the two important enzymes in the formation of the toxic metabolite NAPQI from APAP- were downregulated in mice fed with fructose compared to the levels in mice on a regular chow diet^[52]^. Cho *et al*. also performed enzyme assays using probe drugs (7-ethoxyresorufin for CYP1A2 and chlorzoxazone for CYP2E1), demonstrating the correlation between the decreased CYP2E1 and CYP1A2 activity and fructose intake^[52]^. They also detected increased levels of basal GSH in high-fructose diet-fed mice *vs*. chow-fed mice^[52]^. We also reported significantly higher GSH levels in fructose-fed mice compared to control mice just 1 h after APAP injection. Additionally, mice gavaged with fructose as early as 45 min after APAP overdose showed a 90% reduction in serum ALT and LDH levels compared to mice gavaged with saline. When gavaged with fructose 2 h and 6 h after APAP exposure, the mice still displayed a significant 70% reduction in serum ALT^[53]^. To uncover the mechanism of fructose protection against APAP-induced hepatotoxicity, we investigated the roles of fructose-induced hepatokine FGF21 and its transcription activator: carbohydrate-responsive element-binding protein (ChREBP).

### FGF21 induction by fructose via ChREBP

Fibroblast growth factor 21 (FGF21) is a hepatocyte-secreted hormone that is crucial in the metabolism of glucose and lipids^[54]^. It is expressed primarily in the liver and adipose tissue, significantly enhancing insulin sensitivity and lowering body weight^[55]^. Both hepatic and circulating levels of FGF21 have been shown to be elevated in mice with APAP-induced hepatotoxicity. In addition, this increase in FGF21 expression was observed within 3 h, even before the spike of liver injury markers such as ALT and AST^[54]^. *Fgf21* knockout (KO) mice demonstrated more severe liver damage and oxidative stress compared to wild-type (WT) mice, while adenovirus-mediated overexpression of FGF21 in the liver reversed the injuries in mice. FGF21 induces hepatic expression of the nuclear factor erythroid 2 (NRF2) and peroxisome proliferator-activated receptor γ coactivator 1α (PGC-1α), which are involved in the mammalian response to oxidative stress^[53,54]^.

FGF21 is one of the targets of carbohydrate response element binding protein (ChREBP), a transcription factor that forms a heterodimer with Max-like protein X (MLX) in response to carbohydrate intake^[56]^. It is highly expressed in tissues with high lipogenic activity to activate the expression of lipogenic enzymes^[57]^. ChREBPα, the most common form of ChREBP in hepatocytes, binds to the promoter of target genes through a highly conserved sequence called carbohydrate response element (ChoRE), which was reported in the promoter region of *Fgf21* in both mice (−74 to −52 bp) and humans (−380 to −366 bp)^[57]^.

FGF21 and ChREBP have also been identified as important factors in fructose metabolism. Dushay *et al*. showed that after human patients ingested 75 g of fructose, FGF21 levels reached an average of 3.4-fold increase after two hours^[58]^. Fructose can also upregulate the ChREBP transcriptional activity through post-translational modifications such as phosphorylation, O-glycosylation, and acetylation^[59]^. In our previous report, a high-fructose diet (HFrD) activated hepatic de novo lipogenesis via ChREBP in mice^[60]^.

Our microarray analysis revealed that the expression of *Fgf21* had been altered in WT but not *Chrebp*^*−/−*^ hepatocytes after fructose feeding, prompting us to further explore the stimulation of the ChREBPα-FGF21 axis and its role in the protection against APAP-induced toxicity. Hepatic *Fgf21* mRNA and serum FGF21 levels were suppressed in APAP-injected *Chrebpα-LKO* mice compared to their wild-type counterparts^[53]^. To test whether an increase in FGF21 levels could protect hepatocytes from toxicity caused by APAP, we collected medium from WT hepatocytes transduced with Ad-GFP control, Ad-GFP plus fructose treatment, or Ad-Fgf21. Both Ad-GFP plus fructose treatment and Ad-Fgf21 alone conditioned medium demonstrated half the number of cell deaths compared to the control (< 20% and ~ 45%, respectively), and LDH levels displayed a similar trend^[53]^. Furthermore, the protective effects of fructose were lost in mice with liver-specific deletion of *Fgf21*, while restoration of *Fgf21* expression reversed those effects.

To test whether fructose protects against APAP-induced hepatotoxicity by increasing the expression of FGF21 levels via hepatic ChREBPα, we generated *Chrebpα-LKO* mice by injecting *Chrebpα*^*flox/flox*^ with AAV-TBG-Cre by tail vein whereas the control group was injected with AAV-TBH-GFP. As expected, mice on a high-fructose diet were protected against APAP hepatotoxicity, but deletion of *Chrebpα* resulted in increased necrosis and liver ROS as well as elevated serum ALT and LDH levels^[53]^, confirming that fructose protection against APAP hepatotoxicity is mediated through the ChREBPα-FGF21 axis^[53]^. We and other groups also found that fructose intake decreased the expression levels of two critical enzymes, CYP2E1 and CYP1A2, that catalyze the reaction of APAP to NAPQI^[52,53]^. However, the molecular mechanisms behind the suppression of CYP2E1 and CYP1A2 remain elusive. Both fructose and APAP can induce the expression of FGF21, but so far, little research has been done to tell the difference between APAP-induced FGF21 and fructose-induced FGF21.

## OTHER MOLECULAR PATHWAYS UNDERLYING FRUCTOSE PROTECTION AGAINST APAP-INDUCED HEPATOTOXICITY

Previous studies have shown that FGF21 can regulate metabolic homeostasis in adipocytes by binding to the receptor complex of fibroblast growth factor receptor 1c and β-klotho, activating rapamycin complex 1 (mTORC1) through the MAPK pathway. Minard *et al*. showed that FGF21 induced glucose uptake and stimulated insulin sensitivity in adipocytes through the mTORC1/extracellular signal-regulated kinase (ERK) axis, as reducing the expression of mTORC1 decreased the observed phenotypes^[61]^. In addition, mTORC1 signaling has also been shown to be related to antioxidant mechanisms^[62]^. Other groups reported that mTORC1 can regulate the antioxidant Keap1-NRF2 pathway^[63]^. It is reasonable to speculate that the protective effect of FGF21 against APAP could be mediated through mTORC1. We have already found that overexpression of FGF21 resulted in the increased expression of NRF2 targets such as catalase, *Gst-π*, and *Ho1* without impacting the NRF2 pathway^[53]^. Therefore, it is possible that fructose-induced FGF21 may have other possible targets such as the mTORC1-NRF2 signaling pathway to protect against APAP-induced hepatotoxicity.

Another important aspect of fructose protection against hepatotoxicity was the decrease in levels of CYP2E1. We found that fructose treatment prevented the formation of APAP-protein adducts while maintaining low levels of CYP2E1^[53]^, which is one of the two enzymes crucial for converting APAP into the toxic metabolite NAPQI. Previous studies identified that CYP2E1 was degraded by the endoplasmic reticulum (ER)-associated degradation (ERAD) system, in addition to being a target of the ubiquitin E3 ligase glycoprotein 78 (Gp78)^[64]^. We speculate that fructose might stimulate either ERAD- or Gp78-mediated degradation of CYP2E1 to prevent the buildup of the toxic metabolite NAPQI. Further research is needed to determine the underlying mechanism through which CYP2E1 levels are reduced by fructose.

## CONCLUSIONS AND FUTURE DIRECTION

**Figure 2.**
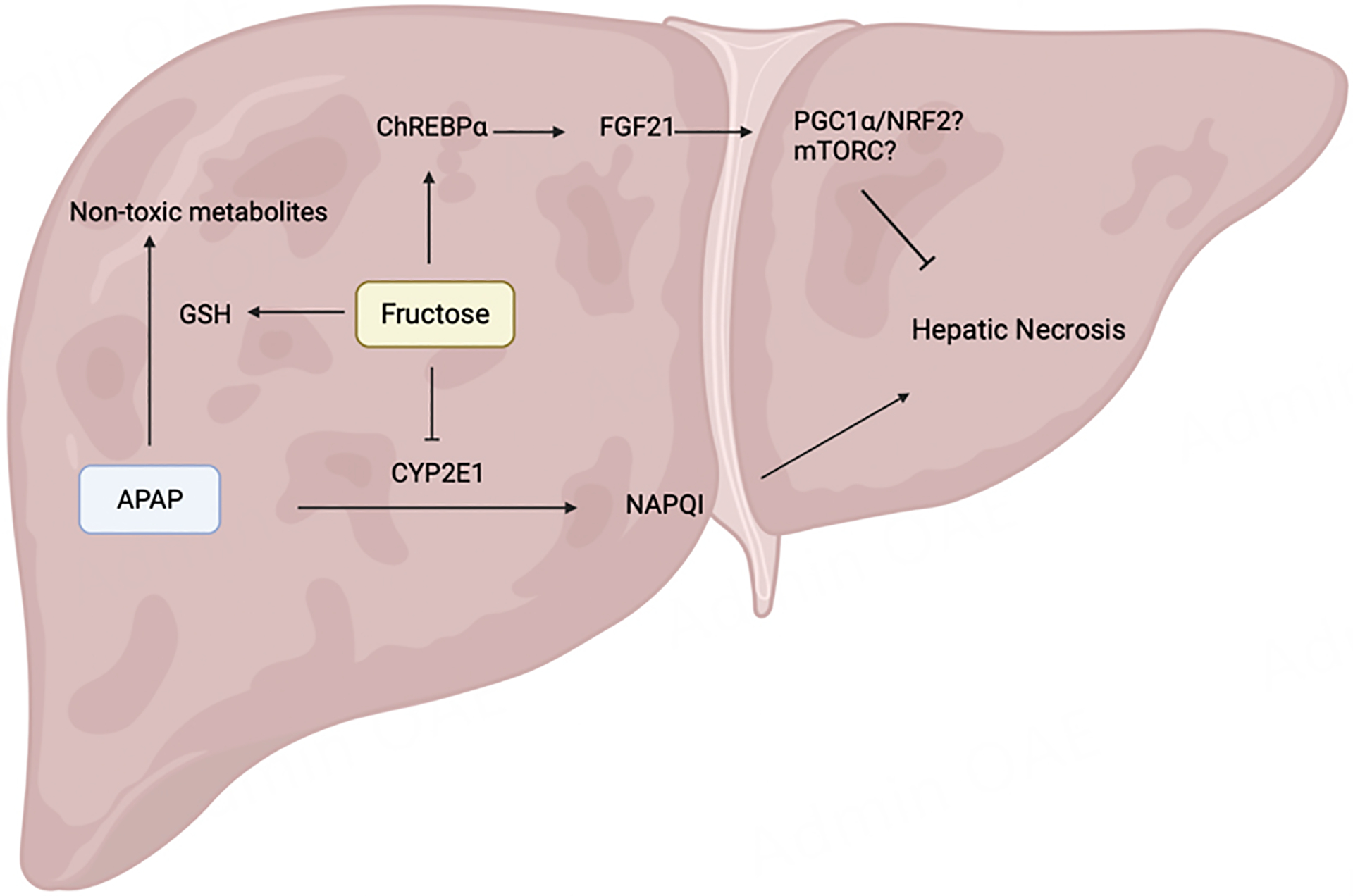
Mechanisms of how fructose protects against APAP-induced hepatotoxicity. APAP: Acetaminophen.

Both published work and our research have revealed that the well-known “bad sugar” fructose has both preventative and therapeutic effects against acetaminophen-induced acute liver injury. Our findings are relatively preliminary. Further research needs to be done using human subjects in order to establish a proper and effective treatment plan. Compared to NAC, fructose might be envisioned as a widely available antidote and first aid in the case of acetaminophen overdose. Based on our current understanding and findings, we summarized how fructose protects against acetaminophen-induced liver damage: (1) by increasing the expression of antioxidant genes through the ChREBPα-FGF21 axis and (2) by regulating factors that are involved in the metabolism of APAP, such as CYP2E1 and glutathione [Figure 2].

Fructose reduces oxidative stress caused by the accumulation of APAP-protein adducts by activating antioxidative genes through the ChREBPα-FGF21 axis. Fructose can also regulate the expression of CYP2E1 and GSH to decrease the rate of conversion of APAP to NAPQI and increase the metabolism of APAP into non-toxic metabolites.

Fructose has been shown to alleviate APAP-induced mitochondrial dysfunction and oxidative stress, two of the major mechanisms underlying most drug-induced liver injury (DILI). It might be intriguing and clinically significant to investigate whether fructose intake could have similar preventative and therapeutic effects against other causes of drug-induced liver injuries, such as those induced by amoxicillin/clavulanate, isoniazid, and nonsteroidal anti-inflammatory drugs^[65]^. Meanwhile, the likelihood that fructose over-consumption might also sensitize the liver to some drug-induced damage should also be accounted. Further research is warranted to provide specific guidance on the use of fructose to treat a variety of drug-induced live injuries.
